# “THEY’VE KIND OF SHUT THAT DOWN WITH COVID”: HOME-LIKE ENVIRONMENTS FOR RESIDENTS WITH DEMENTIA

**DOI:** 10.1093/geroni/igad104.3158

**Published:** 2023-12-21

**Authors:** Phatt Thaitrong, Joy Douglas, Linda L Knol, Amy Ellis, Hyunjin Noh, Shena Sanchez

**Affiliations:** The University of Alabama, Tuscaloosa, Alabama, United States; The University of Alabama, Tuscaloosa, Alabama, United States; The University of Alabama, Tuscaloosa, Alabama, United States; The University of Alabama, Tuscaloosa, Alabama, United States; The University of Alabama, Tuscaloosa, Alabama, United States; The University of Alabama, Tuscaloosa, Alabama, United States

## Abstract

Current guidelines recommend providing a home-like dining environment for older adults living in long-term care (LTC) facilities, as this atmosphere has a positive impact on resident independence, social interaction, mealtime experience, and food intake. In LTC settings, registered dietitians (RDs) play a crucial role in providing nutritional care for residents with dementia. Our study explored RDs’ perspectives on how they provide home-like mealtime environments for LTC residents with dementia. RDs who work with residents with dementia in the U.S. (n = 18) participated in one of five focus groups. Focus groups were led by a trained moderator using a semi-structured interview guide, transcribed verbatim, and audited by the research team for accuracy. Transcripts underwent descriptive content analyses using NVivo© software. Data analyses revealed a significant barrier, the Coronavirus Diseases of 2019 (COVID-19), in providing home-like environments for LTC residents with dementia. RDs reported the negative impact of COVID-19-related isolation among LTC residents with dementia. To mitigate the spread of COVID-19 among vulnerable residents, strict infection control policies were implemented. Therefore, many LTC facilities suspended communal dining, resulting in increased isolation and a sense of social disconnection, exacerbating the progression of dementia in many residents. Thus, COVID-19 presented a significant barrier for RDs to provide a mealtime environment that is home-like for LTC residents. To provide optimal nutrition care for LTC residents with dementia, having a middle road between the COVID-19 policies and traditional practices has been suggested.

